# Computational assessment of the impact of Cu(II) and Al(III) on β-amyloid_42_ fibrils: Binding sites, structural stability, and possible physiological implications

**DOI:** 10.3389/fnins.2023.1110311

**Published:** 2023-02-06

**Authors:** Lorena Roldán-Martín, Mariona Sodupe, Jean-Didier Maréchal

**Affiliations:** Departament de Química, Universitat Autònoma de Barcelona, Cerdanyola del Vallès, Spain

**Keywords:** molecular modeling and simulation, molecular dynamic (MD), amyloid Aβ-42, protein-ligand docking, metal

## Abstract

One of Alzheimer’s disease major hallmarks is the aggregation of β-amyloid peptide, a process in which metal ions play an important role. In the present work, an integrative computational study has been performed to identify the metal-binding regions and determine the conformational impact of Cu(II) and Al(III) ion binding to the β-amyloid (Aβ_42_) fibrillary structure. Through classical and Gaussian accelerated molecular dynamics, it has been observed that the metal-free fiber shows a hinge fan-like motion of the S-shaped structure, maintaining the general conformation. Upon metal coordination, distinctive patterns are observed depending on the metal. Cu(II) binds to the flexible N-terminal region and induces structural changes that could ultimately disrupt the fibrillary structure. In contrast, Al(III) binding takes place with the residues Glu22 and Asp23, and its binding reinforces the core stability of the system. These results give clues on the molecular impact of the interaction of metal ions with the aggregates and sustain their non-innocent roles in the evolution of the illness.

## Introduction

Nowadays, neurodegenerative diseases are hot topics in a broad spectrum of the scientific community. The most predominant one is Alzheimer’s disease (AD) with six out of 10 dementia cases (WHO, 2019). AD first affects the memory areas but as it spreads, other areas of the brain get damaged including those controlling language, reasoning, and cognitive skills. Many efforts have been placed into discovering the onset of the disease and how to reverse it, but no effective treatments have been proposed so far (Breijyeh et al., 2020).

Amyloid plaques and neurofibrillary tangles are the main hallmarks of AD and are caused by the accumulation of β-amyloid (Aβ) and tau protein, respectively (Kepp, 2012; Deture and Dickson, 2019). How these proteins start their aggregation process is still widely unknown. Aβ arises from the cleavage of the amyloid precursor protein (APP) and several hypotheses exist regarding its aggregation mechanism. The most accepted is the amyloid cascade (Hardy and Higgins, 1992), which suggests that the aggregation of Aβ, arising from an imbalance between Aβ production and clearance, is the main causative agent of Alzheimer’s disease pathology. Nonetheless, such aggregation is also observed in healthy patients (Bennett et al., 2006). Another possibility is the metal ion hypothesis (Maynard et al., 2005), derived from the high concentration of metal ions [Zn(II), Fe(III), Al(III), and Cu(II)] in Aβ plaques, which postulates that a loss of metal homeostasis could enhance the aggregation process. Regardless of the onset process, once the aggregation has started, the monomeric forms expand forming oligomers, the most neurotoxic forms, that bind together to form protofibrils and ultimately the entire fibrils. Along all these stages, though, the aggregation unit can be broken producing smaller subunits with seed properties capable of inducing disease in new areas. Accordingly, it is extremely important to properly understand the different phases of the aggregation process to produce effective drugs either to prevent the raise of the disease or slow down its advance (Kepp, 2012).

In the last decades, numerous studies have focused on decoding the folding and aggregation process of Aβ systems (Groh et al., 2017; Almeida and Brito, 2020; Ke et al., 2020; Krishnamurthy et al., 2022; Rahman et al., 2022). Despite these efforts, the elucidation at the molecular level of the aggregation mechanism has not been reached yet. For monomers, the major challenge arises from their intrinsic flexibility, becoming even more complex when they are merged with metal ion binding. To date, several experimental (Drew and Barnham, 2011; Faller et al., 2013; Stefaniak et al., 2021) and theoretical (Huy et al., 2016; Liao et al., 2018; Mutter et al., 2018; Pham et al., 2018; Khatua et al., 2019; Strodel and Coskuner-Weber, 2019; Turner et al., 2019; Boopathi et al., 2020; Roldán-Martín et al., 2021) studies have been performed with respect to different metals (Cu, Zn, and Al) binding to monomeric Aβ. In our previous study, it was demonstrated that monomeric Aβ can adopt a U-shaped structure with two antiparallel α-helix regions, whose secondary and tertiary structure can be modified upon Cu(II) and Al(III) coordination, increasing β-sheet content in the latter case (Roldán-Martín et al., 2021).

However, when it comes to larger aggregated forms such as fibrillary Aβ complexes, the experiments lack the techniques to precisely identify the binding site of metal ions (Miller, 2022). Therefore, there are scarce experimental details on plausible metal-binding sites in fibrils (Parthasarathy et al., 2011), and computational approaches have been shown as suitable techniques to determine the impact of metal binding on Aβ fibril dynamics. Contrary to monomers, aggregate forms are less flexible and several forms of fibrillary structures have been recently reported by X-ray and ss-NMR techniques (Xiao et al., 2015; Wälti et al., 2016; Kollmer et al., 2019; Liberta et al., 2019), providing a solid ground for computational studies to unravel the effect of metal ion binding to amyloid fibrils.

To shed light on those questions, we decided to apply a multi-scale protocol focusing on Cu(II) and Al(III) bound forms of Aβ_42_ fibril, which are known to be more aggregation-prone. The work stands on (1) BioMetAll (Sánchez-Aparicio et al., 2021), a recently released structural predictor of metal-binding sites in proteins, (2) protein-ligand dockings compatible with metallic ligands (Sciortino et al., 2018), and (3) classical and accelerated molecular dynamics. The impact of the metal on Aβ fibril formation and stabilization is assessed with the purpose of providing structural knowledge that could help in understanding the role of metal in the aggregation processes associated with Alzheimer’s disease.

## Materials and methods

**FIGURE 1:**
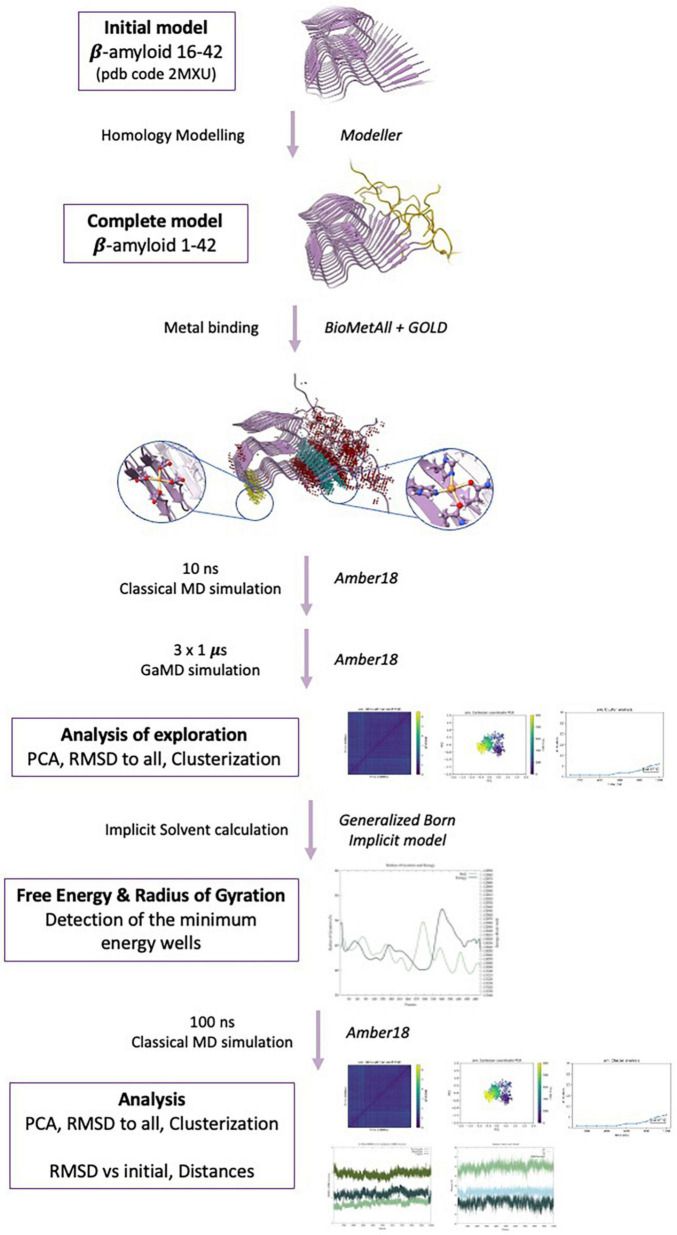
Methodological protocol used in the present study.

The computational protocol followed in this study is summarized in Figure 1.

### Initial models

Several fibrillary structures of the β-amyloid fibril are available in the Protein Data Bank. The most complete PDB structure, with code 2NAO (Wälti et al., 2016), corresponds to the sequence 1–42 of the amyloid for each strand, though it retrieves poor punctuation in all the PDB parameters (clashes score 22, Ramachandran 12.4% and Sidechain 26.7%). On the other hand, the PDB structure with code 2MXU (Xiao et al., 2015) presents far better punctuation (clashes score 0, Ramachandran 3.8% and sidechain 6.22%) at the expense of residues 1 to 11 being missing, an observation consistent with the high flexibility of the N_*Ter*_ region of the peptide. Therefore, for this study, we selected the latest and elongated each strand with the 11 missing residues using homology modeling through the UCSF Chimera Software Modeler tool (Pettersen et al., 2004) to obtain the N-terminal missing region. Chimera minimization was performed to reduce the clashes, with the Amber ff14SB force field (Maier et al., 2015) and 100 steps of steepest descent.

Once the complete fibrillary structure was obtained, several preliminary molecular dynamics calculations were performed. We considered the systems with different numbers of strands, namely 4, 6, 8, and 10 aiming at identifying the minimal number of strands necessary for the fiber to remain stable in the fibrillar form during the trajectory. This is also particularly important to avoid possible drawbacks inherent to force field propensity to stabilize certain secondary structures against others (Case et al., 2005). With the Amber force field, we observed that at least 6 strands are necessary to obtain a fibril, though at least 10 strands should be considered for a stable core. In this study, only the fibrillary structure of 10 strands is discussed.

### Metal-binding areas: BioMetAll and GOLD

Putative metal-binding areas were detected by applying the BioMetAll software (Sánchez-Aparicio et al., 2021) to the Aβ_11–42_ crystallographic structure available in the PDB 2MXU, which was configured to include the backbone atoms and a minimum of three residues as possible coordinating groups. BioMetAll only considers the backbone and the residues that are within a suitable distance to coordinate the metal, but their orientation may not be optimized for coordination. Therefore, protein-ligand dockings calculations were performed with the program GOLD (Jones et al., 1997) using the GoldScore scoring (Jones et al., 1995) function, whose parameter file was modified to include atom types for metal ions and their possible coordinating amino acids (Sciortino et al., 2018). Genetic algorithm (GA) parameters were set to 50 GA runs and a minimum of 100,000 operations each. The remaining parameters, including pressure, number of islands, crossovers, or mutations, were set to default. Finally, docking solutions were analyzed through GaudiView (Rodríguez-Guerra Pedregal et al., 2017) an in-house developed GUI tool built as an extension of UCSF Chimera.

### Classical molecular dynamics simulations for metalloaggregates

Metal parameters were obtained using the MCPB.py package (Li and Merz, 2016) from quantum mechanical calculations with DFT (B3LYP) (Lee et al., 1988; Becke, 1998) and adding Grimme’s D3 correction for dispersion (Grimme et al., 2010). For aluminum complexes, the 6-31 + G (d,p) basis set was used for all atoms (Rassolov et al., 2001). For Cu(II) complexes, we used the 6-31 + G (d,p) basis set for C, H, N, and O atoms; and the SDD pseudo-potential and the corresponding basis-set supplemented with f-polarization function for Cu (Ehlers et al., 1993). Solvent-polarizable dielectric continuum model (SMD) was considered to account for the solvent effects in water (Marenich et al., 2009). Force constants and equilibrium parameters for metal-coordinating atoms were obtained through the Seminarios’s method (Seminario, 1996) while point charges were derived using the RESP (restrained electrostatic potential) model (Bayly et al., 1993).

Molecular dynamics simulations were then performed with AMBER18 (Case et al., 2018) using the AMBER ff14SB (Maier et al., 2015) force field in the NPT ensemble, with a 1 fs integration time step. The initial models were embedded within a cubic box of pre-equilibrated TIP3P (Jorgensen et al., 1998) water molecules and Na^+^ ions were included to balance the total charge depending on the system: 30 for the metal-free system, 20 for the copper-bound system, and 15 for the aluminum-bound system. Constant temperature and pressure were set by coupling the system to a Monte Carlo barostat (Duane et al., 1987) at 1.01325 bar and a Langevin thermostat at 300 K (Loncharich et al., 1992). The SHAKE algorithm (Ryckaert et al., 1977) was used to constrain the bonds involving hydrogen atoms. Classical MD simulations were performed for 10 ns for all the systems, just to equilibrate the system before running Gaussian accelerated molecular dynamics (GaMDs). Moreover, MD simulations have also been performed on the lowest energy wells obtained in the GaMD simulations.

### GaMDs simulations

Enhanced conformational sampling was performed with the Gaussian accelerated molecular dynamics (GaMDs) (Miao et al., 2015). For these simulations, the AMBER ff14SB force field in the NVT ensemble was used, constraining the bonds involving H atoms with SHAKE, with an integration time step of 2 fs. A boost on both dihedral and total potential energy (igamd = 3) was applied. In total, three GaMD replicas of 1 μs were produced for each system.

### Analysis

The energy of the systems was calculated along the trajectory with the Generalized Born Implicit model (Onufriev et al., 2000, 2004), for 1,000 frames extracted from each 1 μs GaMD trajectory, stripping water molecules, and performing a short minimization (maxcyc = 500 steps) previously to the energy extraction. The radius of gyration (RoG) was computed over the same 1,000 frames, so a graphic with the different minima was obtained. For each system, the well with the minimum energy was selected for an MD, to test its stability, and for the analysis.

Principal component analysis (PCA) (Wold et al., 1987), RMSD all-to-all, and clustering were performed over both the GaMD and MD simulations to test whether the exploration was exhaustive enough. For the MD simulations performed on the lowest energy wells from the GaMDs, the RMSD with respect to the initial structure was also computed. The last analysis performed consisted of studying the PCA movements of the fibers along the trajectories with the VMD tool NMWiz and counterchecked by normal mode analysis performed with WEBnma software (Humphrey et al., 1996; Bakan et al., 2011) to study the global low-energy internal motions of the systems.

## Results and discussion

This work aims at investigating the effect of the binding of Cu(II) and Al(III) to the Aβ_1–42_ fibers through molecular modeling. We first present the study of the metal-free fiber to generate a point of comparison and then pursue metal-bound systems.

### Dynamical behavior of metal-free fibril

**FIGURE 2:**
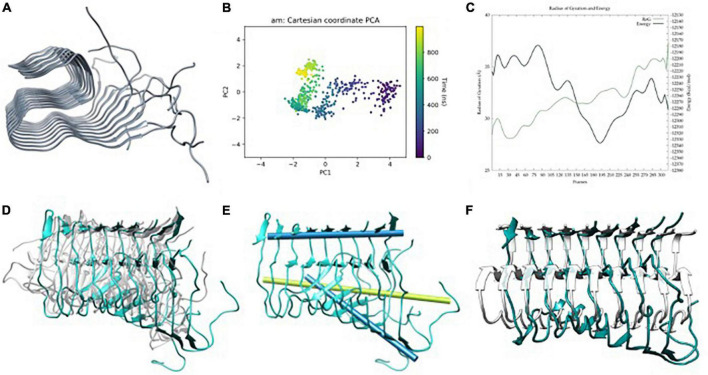
**(A)** Aβ_42_ model built from Aβ_11–42_ (2MXU) (Xiao et al., 2015) after adding the 1–11 residues, **(B)** principle component analysis (PCA) of Gaussian accelerated molecular dynamics (GaMD) simulation, **(C)** energy profile and radius of gyration (RoG) along the GaMD simulation, **(D)** overlap of representative structures of most populated clusters obtained from GaMD simulations, **(E)** in blue, metal-free fibril axis representation of 39–41 (top) and 11–15 β-sheets (bottom), in green; 11–15 β-sheets axis of NMR-structure, and **(F)** overlap between the solid-state NMR (ss-NMR) structure and the lowest energy structure of the GaMD simulation.

The initial structure of the metal-free fibril has been obtained from the solid-state NMR (ss-NMR) structure of Aβ_11–42_ (pdb code 2MXU). Since the N_*Ter*_ end of the system is missing (residues 1 to 10), the structure was completed by homology modeling using modeller (Martí-Renom et al., 2000; Figure 2A). Then, long GaMD simulations were performed using a biased potential and the results were analyzed using several statistical tools.

The total 3 μs GaMD simulation spread over three replicas of 1 μs. The three replicas exhibit similar behaviors in terms of global breathing motions and exhaustive exploration of the conformational space. Accordingly, PCA and energetic profiles of the replica with the lowest energetic conformation identified are presented in the main text (Figures 2B, C) while the other ones are reported in ESI (Supplementary Figure 1). Principal component analysis shows a displacement of the system at the end of the first 100 ns, then, it explores a limited region of the space until approximately 600 ns, and finally, it explores back the previous region, so that the simulation is converged (Figure 2B). The potential energy profile clearly indicates that during the trajectory, a series of low-energy wells are explored with a radius of gyration slowly raising–meaning a geometric expansion of the system (Figure 2C). This is in line with the results reported by Miller et al. (2012), who suggest that fibers do not show a unique lower energy state but different conformers with barriers between them, which are expected to be more or less favorable upon metal binding.

The trajectories present a series of interesting features. The first one is that only the six central strands maintain the fibrillary structure while the two at each extreme tend to unfold. This is related to the fact that these extremes are more exposed to the solvent and naturally start to adopt a more molten globule geometry with some helical contents, a situation reminiscent of the simulations of monomeric peptides. The second is that the terminal regions of each strand represented by the amino acids from 1 to 10 are overall disorganized. This is consistent with the absence of atomic density in the pdb structure and suggests that the overall geometry of the amyloid is barely affected by the N_*Ter*_ region adopting quite random conformations. Based on this, the N_*Ter*_ region and the four strands at the extremes are discarded for the rest of the analyses, which focuses on the core formed by the six central strands and from residues 11 to 42.

The major motion observed is a fan-like movement of the Aβ_11–20_ β-sheet, which appears to be the natural breathing movement of the system (Figure 2D) as counterchecked by normal mode analysis performed using WEBnma software (Tiwari et al., 2014). This fan-like motion consists of a hinge that can be defined as the angle between the principal axes of the sets of atoms of residues 11 to 15 and 39 to 41 (Figure 2E). The angle obtained between these two principal axes for a representative structure of the most populated cluster reaches 48.5° against the 7.8° of the ss-NMR structure. For the lowest energy structure, the same analysis shows a hinge of 46.7° (Figure 2F), which still represents the main distortion with respect to the ss-NMR structure. These simulations and analyses show that the metal-free fiber mostly retains the experimental geometry although it can display the dynamic motions not observed in the experimental structures.

### Predicting metal-binding sites in amyloid aggregated structures

The binding of metal ions to amyloids has been the source of an extensive number of studies (Faller, 2009; Mutter et al., 2018; Pham et al., 2018; Roldán-Martín et al., 2021; Miller, 2022). Today, a relatively clear picture of metal binding has emerged in the monomeric systems, either theoretically for Al(III) (Mujika et al., 2017) or experimentally for Cu(II) (Drew and Barnham, 2011), which has narrowed the location and type of amino acids that bind the metal ions with only few candidates, all part of the N_*Ter*_ region of the peptide. For aggregates, though, there is still a substantial level of uncertainty.

The previous part of our study sustains an intrinsically disordered protein (IDP) behavior of the N_*Ter*_ region of the aggregate. This flexibility indicates that the binding of the metal ions to this region would be similar to what occurs in monomeric species. It also shows that the core of the aggregate is quite rigid, and one would naturally wonder if this rigidity induces some pre-organizations for metal binding.

To identify putative metal-binding sites in the aggregates, we performed BioMetAll calculations, a software recently released by our group for the prediction of metal-binding sites in biomolecules. Based on the backbone pre-organization hypothesis, BioMetAll has shown excellent success rates in identifying metal-binding locations for a wide sample of proteins and ions (Sánchez-Aparicio et al., 2021). To search for site-specific binding areas for copper and aluminum, the search was first performed looking for a minimum of three coordinating amino acids and any amino acid known to participate in the coordination sphere of transition metals. With this screening, BioMetAll finds the geometries compatible with the simultaneous coordination of two His residues in the N_*Ter*_ region of the Aβ fibril as identified in the monomers (Alí-Torres et al., 2011, 2014) from the experiments and QM calculations. However, this only occurs in a few strands due to the intrinsic flexibility of that region. Since other metal-binding sites are more frequently found in the core region of the fibril, indicating that in this system, the situation is different from what happens in monomers, we discarded N_*Ter*_ monomer-like binding sites and focused on the core region, favoring Asp and Glu for Al(III) (Mujika et al., 2017) and His and Gln for Cu(II). For the latter, it is important to notice that Gln residues have been included in the search since, although rare, the coordination of Gln to Cu(II) is observed in a series of X-ray structures from the protein data bank, it has also been proposed as a possible coordinate residue (Parthasarathy et al., 2011), and Gln15 shows the proper location for metal coordination in the selected structures.

**FIGURE 3:**
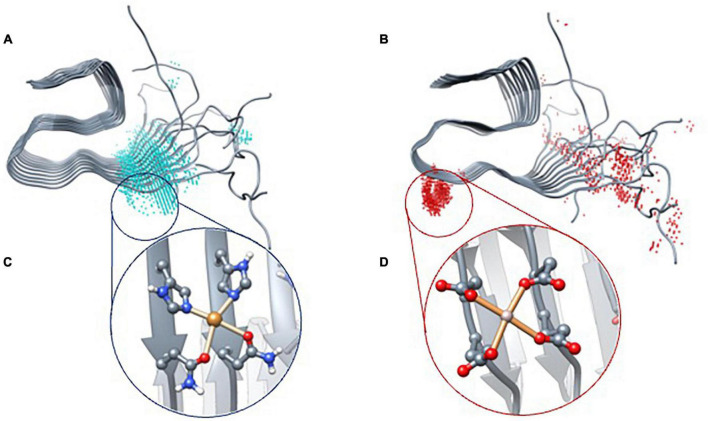
BioMetAll results of Cu(II) **(A)** and Al(III) **(B)** coordination. Once the areas were identified, docking results with Gold software were obtained for Cu(II) **(C)** and Al(III) **(D)**.

In total, two areas were found for metal binding in the core of the fibril: one consistent with copper and the other with aluminum. The former, named Cu_*F*_, stands at the linker between the N_*Ter*_ loop and the β-strand regions (from residue 12–15) and is composed of histidine His13 of two adjacent strands and two glutamine Gln15 of the same strands (Figure 3A). The latter, named Al_*F*_, is located at the beginning of the coil with residues Glu22 and Asp23 of adjacent strands (Figure 3B). This latter site agrees with the work reported by Yang et al. (2022) which shows that this area is likely to be bound to a metal ion. With these two sites identified, we pursued generating realistic three-dimensional models of the metal-aggregate systems by carrying out protein-ligand dockings. Those were performed with GOLD, assuming a square planar environment for Cu(II) and an octahedral one for Al(III) (Figures 3C, D).

The results indicate that Cu(II) can achieve a square planar coordination with two His and two Gln from adjacent strands. However, for Al(III), docking results only find an incomplete octahedral coordination in which two Glu and two Asp of adjacent strands are involved, thereby leaving vacant sites that would probably be occupied by the solvent molecules. Each model was then submitted to triplicated Gaussian accelerated molecular dynamic simulations to evaluate its stability and dynamics properties as well as to compare with the metal-free system.

### GaMD of metal bound fibers

**FIGURE 4:**
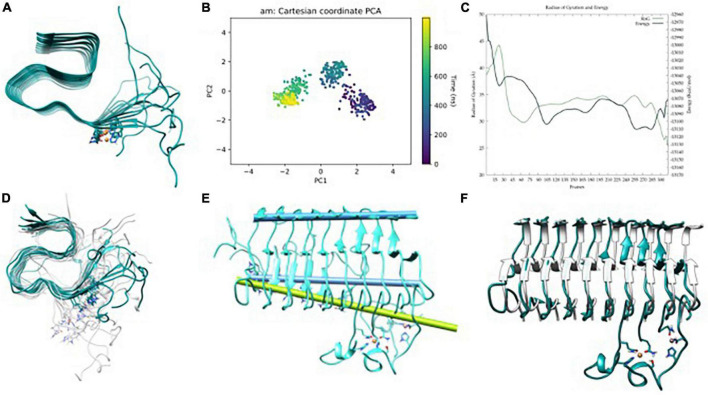
**(A)** Aβ_42_-Cu(II) model, **(B)** principle component analysis (PCA) of Gaussian accelerated molecular dynamics (GaMD) simulation, **(C)** energy profile and radius of gyration (RoG) along the GaMD simulation, **(D)** overlap of representative structures of most populated clusters obtained from GaMD simulations, **(E)** in blue, axis representation of 39–41 residues (top) and 13–16 β-sheets (bottom); in green; 13–16 β-sheets axis of NMR-structure, and **(F)** overlap between the solid-state NMR (ss-NMR) structure and the lowest energy structure of the GaMD simulation.

Data of the triplicated simulations on Cu_*F*_ are given in Figure 4 and Supplementary Figure 2 of ESI. In all cases, the trajectories tend to converge around 400 ns (Figure 4B and Supplementary Figures 4D–F) and show the presence of a series of minima (Figure 4C). Such minima agree with the fact that amyloids are highly polymorphic, metal binding shifting the population toward a certain conformation (Miller et al., 2010). Overall, the 3 μs GaMD simulations depict the same fan-like motion observed in the metal-free fiber (Figure 4D). Strikingly though, the geometry variability of the N_*Ter*_ loop (residues 1–11) is significantly more pronounced in Cu_*F*_; i.e., each metal-bounded pairs of strands have their motions uncoupled from adjacent pairs. Consequently, the β-sheet H-bonds between these adjacent pairs are significantly weakened, which favors the change in their secondary structure increasing the α-helix content in the N_*Ter*_ ends, particularly on the first strands. Such α-helix regions are reminiscent of the monomeric forms, a behavior more pronounced than in the metal-free system (Roldán-Martín et al., 2021). However, the hinge movement previously observed in the metal-free fiber appears to be reduced, the angle between the two principal axes (considering the six central strands as done previously for the metal-free fiber) in a representative structure of the most populated cluster being 6.1° (Figure 4E). Note that in this case, the motion is not globally spread all other the fibril since there is a disruption of the fibrillar form that arises from a higher displacement of the first strands with respect to the remaining ones, probably due to the repulsion between Cu(II) ions (Figure 4F). Such observations may indicate that Cu(II) binding tends to disrupt the fibrillar form to lower-aggregation structures or amorphous aggregates without fibrillary structure.

**FIGURE 5:**
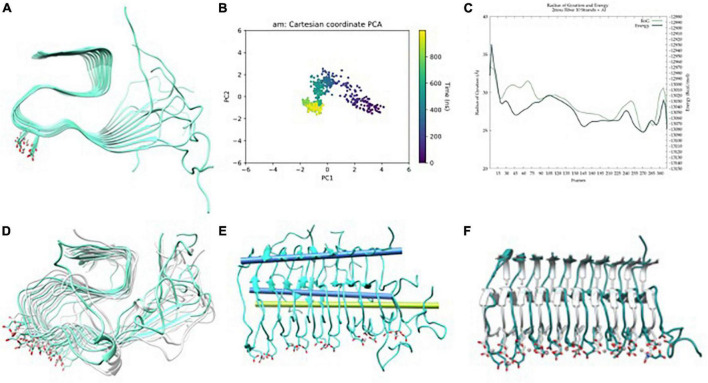
**(A)** Aβ_42_-Al(III) model, **(B)** principle component analysis (PCA) of Gaussian accelerated molecular dynamics (GaMD) simulation, **(C)** energy profile and radius of gyration (RoG) along the GaMD simulation, **(D)** overlap of representative structures of most populated clusters obtained from GaMD simulations, **(E)** in blue, axis representation of 39–41 residues (top) and 13–15 residues β-sheets (bottom); in green; 13–16 β-sheets axis of NMR-structure, and **(F)** overlap between the solid-state NMR (ss-NMR) structure and the lowest energy structure of the GaMD simulation.

For Al(III), the preferred binding site in the fiber involves the residues Glu22 and Asp23 of adjacent strands (Figure 5A). In this last case, the GaMD explores a series of minima until finding a well it cannot escape during the last 400 ns (Figure 5B and Supplementary Figure 3). Despite the energy profile highlighting a remarkable minimum (Figure 5C), the whole trajectory displays a very stable fold with minor motions (Figure 5D). In particular, the amplitude of the fan-like movement is far more limited in Al_*F*_ than in the other two systems. For example, one of the most representative structures presents an inter-axial angle of only 2.7°, similar–even lower–than that in the ss-NMR structure (7.8°) (Figures 5E, F). Therefore, GaMD simulations show that the aluminum-bound aggregate displays a more compact structure than both free-metal and copper-bound ones and remains closer to the static view provided by experimental ss-NMR geometry (Figure 5F).

### Further analysis

To further analyze how the metal ions impact the conformation landscape and stability of the aggregates, classical MDs of 100 ns were carried out starting from the structure of the lowest energy well obtained from the GaMD simulations. To test the exhaustiveness of the exploration and their stability, RMSD and PCA were performed on the trajectories (Supplementary Figure 4). Emphasis is given to how metal binding alters the fiber’s low collective modes and breathing motions. For that, five distances were evaluated using the carbon α of each residue and considering only the six central strands of the fibril. To ease the labeling and the reading, we use the X-ResNum identification format where X is the number of the strand the residue belongs to, Res is the type of the residue, and Num is its number in the sequence.

**FIGURE 6:**
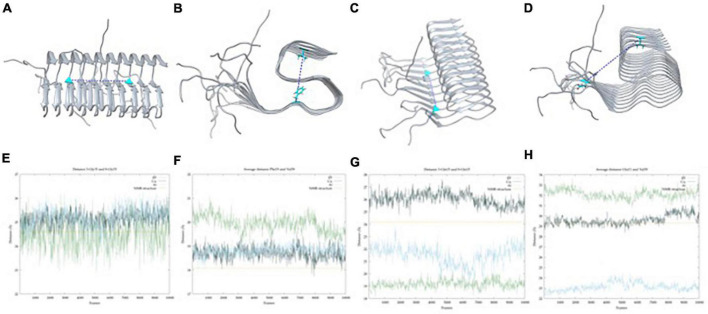
Atoms chosen for the horizontal distance **(A)**, vertical distance **(B)**, 11–15 β-strand intersheet distance **(C)** and opening of the 1–18 region **(D)** represented in the solid-state NMR (ss-NMR) model. Results of the horizontal **(E)**, with an average distance of 24.6 (± 5.6)Å, 25.2 (±1.6)Å and 25.5 (±10.9)Å for metal-free, Cu(II) and Al(III) systems; vertical **(F)**, with an average distance of 19.9 (±0.3)Å, 18.7 (±0.2)Å and 18.8 (±0.2)Å for metal-free, Cu(II) and Al(III) systems; intersheet **(G)**; with an average distance of 19.3 (±5.2)Å, 26.06 (±1.1)Å and 21.5 (±0.7)Å for metal-free, Cu(II) and Al(III) systems; and opening **(H)**, with an average distance of 32.1 (±0.4)Å, 29.6 (±0.5)Å and 23.1 (±0.4)Å for metal-free, Cu(II) and Al(III) systems; along the MD simulation, with ss-NMR distance represented in yellow line.

**FIGURE 7:**
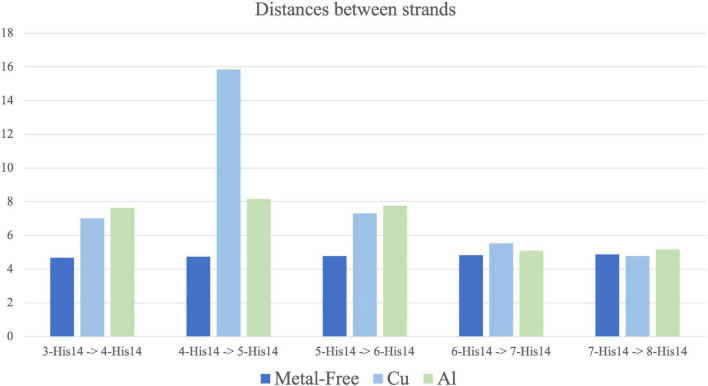
Average distance between His14 from each core strand to the adjacent one along 100 ns molecular dynamic (MD) simulation for metal-free fibril (dark blue), Cu(II) (light blue) and Al(III) (green). Standard deviation range from 0.1 to 0.7. Strands 3–4, 5–6, and 7–8 are bound by metal coordination in Cu(II) and Al(III) systems.

1. The distance between 3-Gly33 and 8-Gly33 measures how the fiber opens horizontally in the core region and indicates if the β-sheet interaction is weakened (longer distance) or strengthened (shorter distance) (Figure 6A).
2. The average distance from Phe19 to Val39 of each core strand indicates how the fiber opens vertically and measures the compactness of the S-shaped supramolecular structure (Figure 6B).
3. The distance between 3-Gln15 and 8-Gln15 measures how the fiber opens horizontally in the N_*Ter*_ region and is indicative of the β-sheet interaction at this region (Figure 6C).
4. The average distance from Glu11 to Val39 of each core strand describes the separation movement of the N_*Ter*_ tail from the core region of the fiber (Figure 6D).
5. The average distance between His14 of each core strand describes if the fiber movement is collective (equal distance between strands) or individual (Figure 7).

With these descriptors, it is possible to see that the three complexes behave similarly both in the horizontal and in the vertical axes (measures 1 and 2). For measure 1 (Figure 6E), the metal-free fiber shows a distance of 24.64 (±5.6) Å, while Al(III) and Cu(II) exhibit values around 25 Å, which are very similar to the 24.6 Å of the ss-NMR structure. Distances of measure 2 (Figure 6F) are also similar, with the values of 19.9 (±0.3) Å for the metal-free fiber, 18.7 (±0.3) Å and 18.8 (±0.2) Å for the Cu and Al bound complexes and 18.3 Å for the ss-NMR structure, respectively. The other distances, though, demonstrate the substantial differences between the metal-free fiber and the metal-bound complexes.

Measure 3 relates to the disruption of the fibrillary structure in the N_*Ter*_ region. It is observed that Cu(II) coordination produces an increase in this distance, from 24.3 Å in the ss-NMR to 26 (±1.1) Å. In contrast, both the metal-free fibril and Al(III)-bound structure show shorter distances, of 19.93 (±0.3) Å and 21.5 (±0.7) Å, respectively, in comparison with the ss-NMR structure (Figure 6G). Measure 4, on its side, is remarkably increased in the metal-free fibril [32.1 (± 0.4) Å] with respect to the ss-NMR (29.3 Å), due to the hinge fan-like movement. Cu(II) system is maintained in a similar value to the ss-NMR structure, with a distance of 29.6 (±0.5) Å. Note, however, that this distance is an average of six values and the displacement of the terminal strands is faded by the shorter distance retrieved by the other ones. Indeed, the two most external strands included in the average exhibit values of 35 and 42 Å, respectively, whereas the four inner ones show values that range between 20 and 25 Å. Finally, the Al(III) system is even more compact than the ss-NMR structure, with an average distance of 23.1 (±0.4) Å, supporting the fact that aluminum binding increases the stability of the fibrillary conformation (Figure 6H).

The distances between each pair of strands (measure 5) were also measured (Figure 7). Calculations confirm the trends previously observed. The metal-free fibril has approximately the same distance between each pair of strands. Such system has a higher flexibility on the vertical axis, as measures 2 and 4 also support, while the horizontal axis is more compact and shows a collective movement of all the strands, which corresponds to the hinge fan-like movement. However, upon metal coordination, the distance between the strands differs.

First, Cu(II) ion, which binds to the N_*Ter*_ region, increases the distance between each pair of strands, even between those bound to the same metal ion. The most remarkable is the increase in the 4-His14–5-His14 distance, which indicates a disruption in the collective movement of the strands (Figure 7). This only happens in the Cu-bound system. For Al(III), the distance between the pairs of strands is also increased, especially in strands 3 to 6, but the collective movement is preserved. This points to the fact that, although Al(III) binding increases the compactness and stability of the core region, as measures 1 to 4 support, the (N_*Ter*_) region is still highly flexible.

**FIGURE 8:**
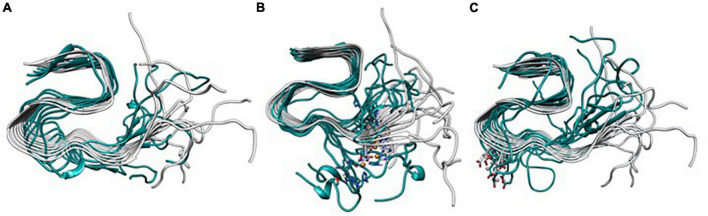
Overlapping of the lowest energy structure obtained from the Gaussian accelerated molecular dynamics (GaMD) simulation of Metal-Free Fibril **(A)**, Cu(II) **(B)**, and Al(III) **(C)** against the Aβ_42_ model.

The mentioned changes in metal coordination are also clearly observed if the systems are overlapped against the ss-NMR structure (Figure 8), which demonstrates that the metal-free fiber has a collective hinge fan-like movement, increasing the flexibility of the system but without its disruption. In contrast, Cu(II) leads to the partial dismantling of the fibrillary conformation in the (N_*Ter*_) region by breaking the collective movement of the fiber. Finally, Al(III)-bound system is more compact in the core region, though the (N_*Ter*_) region is still highly flexible.

## Conclusion

One of the Alzheimer’s disease main hallmarks is the formation of amyloid plaques, constituted by the aggregates of β-amyloid peptides. In this process, metal ions have been shown to play an important role. In the present study, an integrative computational study has been performed to unravel the role of Cu(II) and Al(III) ion binding in the β-amyloid fibrillary structure (pdb code 2MXU). To do so, the binding site and the coordination sphere of the metallic ions were identified with a combined protocol of BioMetAll and Gold Software. Once binding sites were properly detected and the tridimensional models properly set up, the complexes were submitted to an exhaustive conformational exploration through MD and Gaussian accelerated MD, with three replicas of 1 μs each, achieving a total of 3 μs for the metal-free fiber, Cu(II)-bound, and Al(III)-bound systems. The resulting simulations were analyzed and compared between them and in front of the initial ss-NMR structure.

Metal-free simulations allowed us to characterize the collective low-energy vibrational modes that constitute the natural breathing motions of the S-shaped structure. The main one could be described as a hinge fan-like movement due to the opening–closing tendency of the (N_*Ter*_) region of the fiber. Such movement could be linked to the dynamic process of aggregating and dismantling β-amyloid fibers. Metal-bound complexes show a differential behavior that, interestingly, depends on the metal ion. On the one side, Cu(II) binding is at the (N_*Ter*_) region with two His13 and Gln15 from adjacent strands, at a site very close to where it happens in the monomeric species. The binding of copper ion introduces the charges in the (N_*Ter*_) region and the analyses of the simulations show that the system tends to disrupt the fibrillary structure of the most extreme strands that adopt α-helix configurations, resembling the monomeric structure of the β-amyloid peptide. This fact supports the hypothesis that copper is more prone to lead to amorphous aggregates (Huy et al., 2016), as shown experimentally (Innocenti et al., 2010). In contrast, Al(III) binding takes place in the first coil region of the S-shape, on the residues Glu22 and Asp23 from adjacent strands, which compensates for the Al(III) charge. In this case, aluminum ion binding reinforces the stability of the system in the core region, even reducing the hinge movement observed in the metal-free fiber complex and obtaining structures with the lowest difference with respect to the ss-NMR structure. Such fact reinforces the idea that Al(III) stabilizes neurotoxic species (Miller et al., 2012). In contrast, the larger stability of the fibril has been related to a higher aggregation rate (Thu and Li, 2022). However, mature fibril deposits are less relevant than oligomers with respect to AD etiology and severity (Miller et al., 2012).

Overall, the computational study carried out in this work shows that the binding of metal ions to β-amyloid fiber indeed affects its dynamical behavior, though in a different manner depending on the metal ion involved: while Cu(II) leads to less organized tertiary structures than the unbound system, Al(III) retrieves the opposite behavior, with a high stable S-shaped structure. This is in line with the study of Bolognin et al. (2011), which demonstrates that while Cu(II) prevents the formation of fibrillary aggregates, Al(III) induces the aggregation of fibrillary oligomers. The next steps are being performed to merge the influence of metal-ion coordination with the relevant familiar mutated forms of the β-amyloid fiber.

## Data availability statement

The original contributions presented in this study are included in this article/Supplementary material, further inquiries can be directed to the corresponding authors.

## Author contributions

LR-M carried out the experiment. LR-M, J-DM, and MS wrote the manuscript. J-DM and MS supervised the project and conceived the original idea. All authors contributed to the article and approved the submitted version.
