# Supplementary material for: Computational assessment of the impact of Cu(II) and Al(III) on β-amyloid42 fibrils: Binding sites, structural stability, and possible physiological implications
Source: Front Neurosci. 2023 Feb 6;17:1110311. doi: 10.3389/fnins.2023.1110311 (PMC9940836; doi:10.3389/fnins.2023.1110311)
Supplement: Supplementary file 1 [file Data_Sheet_1.pdf]

Supplementary Information: Computational Assessment of the Impact of Cu(II) and Al(III) on β–Amyloid$_{42}$ Fibrils: Binding Sites, Structural Stability and Possible Physiological Implications

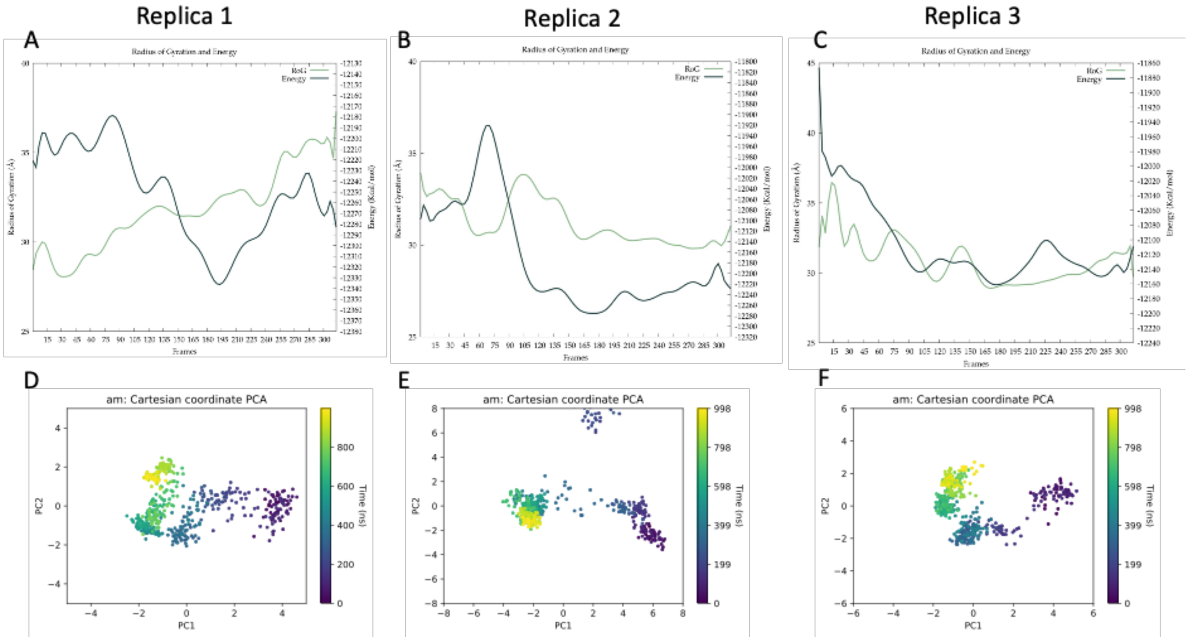

Supplementary Figure 1. A, B and C. Energy profile and RoG along the GaMD simulation along the three replicas of the metal free fiber. D, E and F. Principle Component Analysis (PCA) of the same simulations.

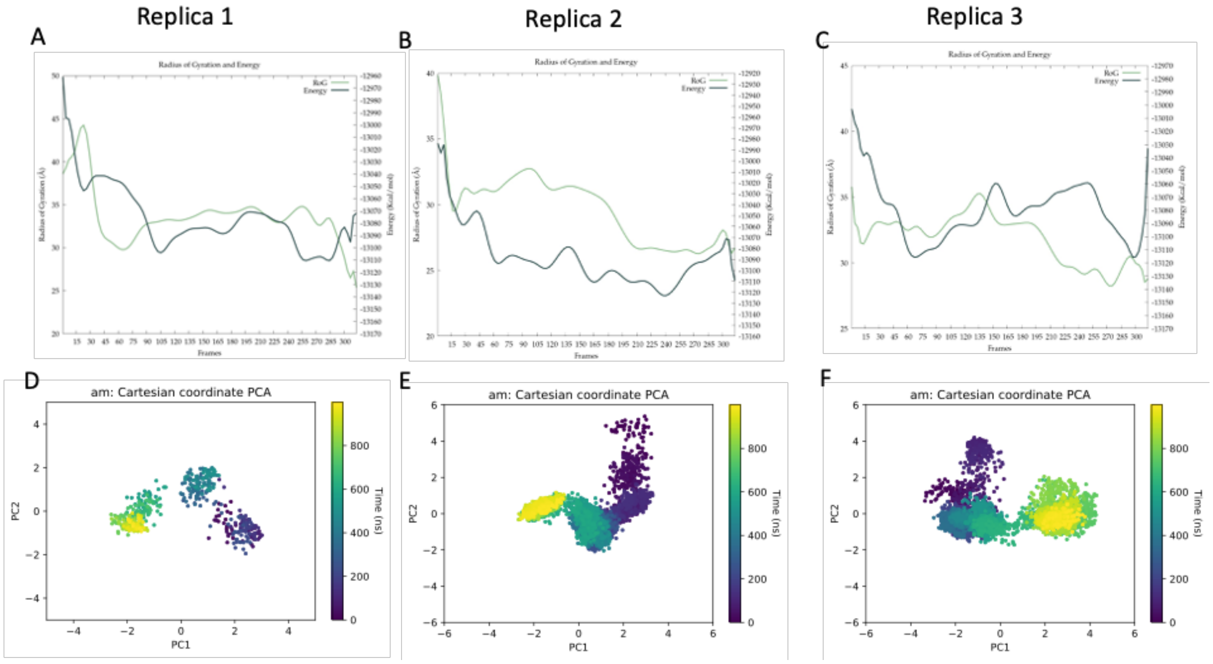

Supplementary Figure 2. A, B and C. Energy profile and RoG along the GaMD simulation along the three replicas of the Cu(II) binding fiber. D, E and F. Principle Component Analysis (PCA) of the same simulations.

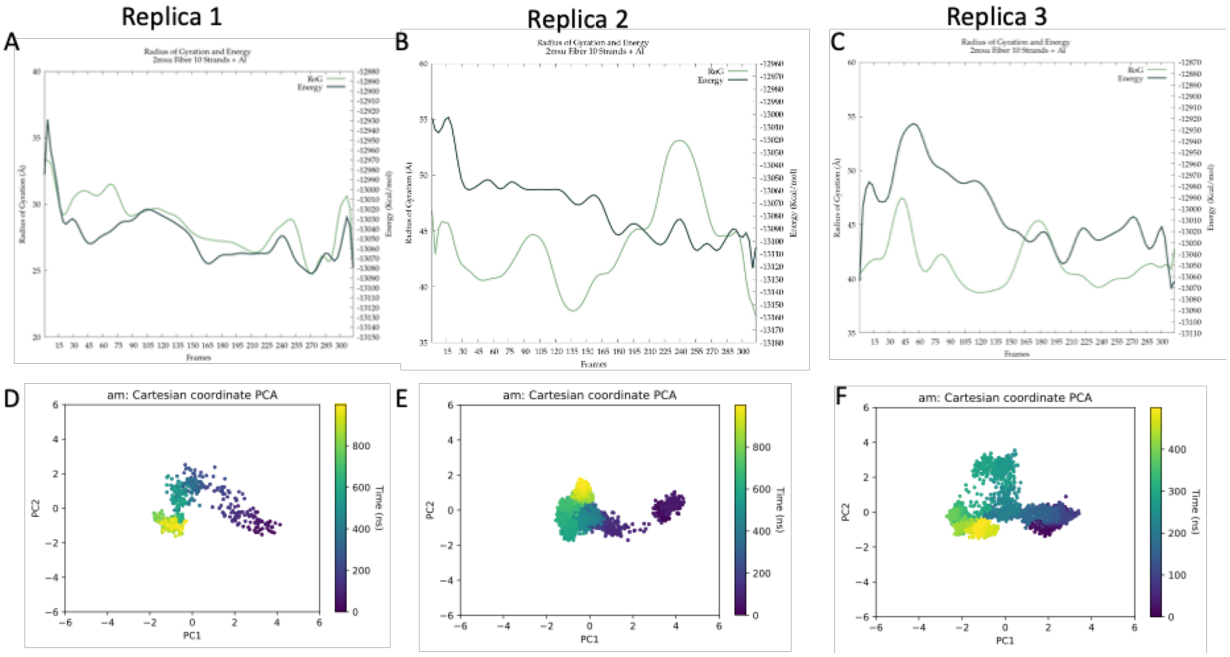

Supplementary Figure 3. A, B and C. Energy profile and RoG along the GaMD simulation along the three replicas of the Al(III) binding fiber. D, E and F. Principle Component Analysis (PCA) of the same simulations.

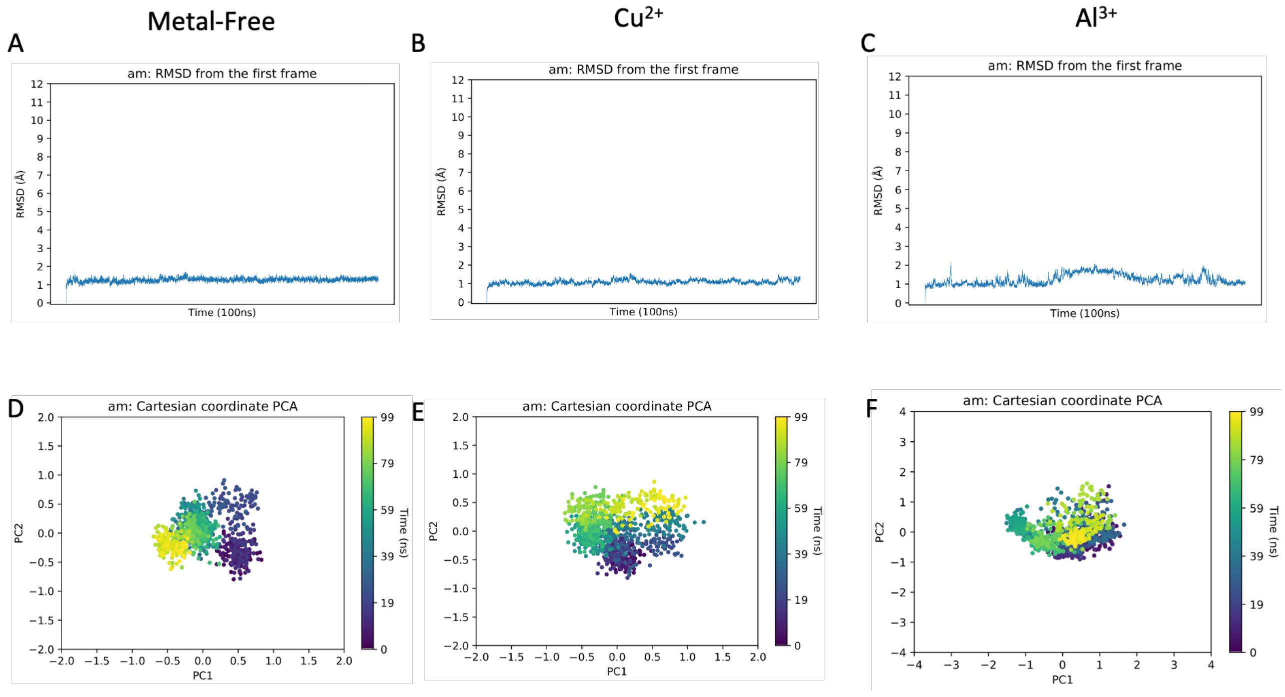

Supplementary Figure 4. Stability analysis for classical MDs from the structure on lowest energy well obtained in GaMD simulation. RMSD and PCA analysis performed over 100 ns trajectories of Metal-free system (A and D), Copper-bound system (B and E) and Aluminium-bound system (C and F).
